# Correspondence

**Published:** 1906-08

**Authors:** T. M. McIntosh

**Affiliations:** Thomasville, Ga.


### CORRESPONDENCE.

---

Mr. Editor: Your issue of June has just reached me. I have read your editorial, "Not to be Alligned," and from it I draw the conclusion that some of those who have been, and are, prominent in the so-called "reorganization" of the Medical Association of Georgia have made verbal or written protest because you published "a letter from one of our (your) friends, medically, who refused to go into the reorganization." Your editorial recalls to mind an editorial in the *Georgia Practitioner,* published a short while, I think, before the Atlanta meeting, the gist of which, as I remember it, was that when the reorganization became an accomplished fact, that the Medical Association of Georgia would select some one of the medical journals, now published in Georgia, as the "organ" of the Association which would give the publication so chosen such prestige of circulation and influence that other journals could not continue to be published; and that he accepted it as a foregone conclusion that he would not be able to continue publication of his journal were it not chosen by the Association. His editorial was not in the spirit of criticism, but of willing and necessary acceptance of conditions.

This *Practician* editorial is not at hand, and if its general meaning has not been correctly stated, I trust the error will be pointed out and that this allusion to it will be accepted as purely impersonal, and only as a link in a chain of evidence to show the correctness of some conclusions at which I have arrived and hope to make evident to others as they are to me.

Kenneth W. Millican, M.D., of St. Louis, *Medical Record, July* 7, 1906, says:

"*The California State Journal of Medicine,* in its issue of January, 1906, quoted approvingly a statement from the *Journal of Medical Society of New Jersey,* that: "The day of the privately owned medical journal is passing away. Its place will be taken by the *Journal of the American Medical Association* and the various State journals." Also: "The pages of the official organ (meaning the *Journal of the A. M. A.*) will be controlled by those who control the organization itself, and since the admitted purpose is that it shall be the only medium of communication between members of the profession, there will be no opportunity for the malcontents, however numerous, to get together and organize in opposition to the Oligarchy." Dr. Hicks, in a brochure which he sent out to the profession of Georgia before the Atlanta meeting, and in opposition to the plan of reorganization, shows where the A. M. A. declined to allow Dr. H. B. Young, of Burlington, Ohio, to read a paper before that body which was already on the published program, because he refused to go into the State reorganization.

In May of this year there was mentioned in a daily paper that a movement was being made by a number of physicians of the State to form a new State Medical Society. In the *Atlanta Constitution* of May 20th Dr. Lyle, of Augusta, Ga., is quoted at length as being "bitterly opposed" to the formation of any new State Society, characterizing those who are favoring the movement as a "few disgruntled politico-members" and declares that no other "State society can be formed, because the present one is a chartered body." Incidentally will say that Dr. Lyle would be sure to lose his case at law, and that the present constitution of the present State Association could be legally declared void.

Dr. Jno. C. Olmsted, of Atlanta, Ga., in the *Savannah News* of May 25th, also opposes the proposed new Association, and also speaks of them as former members who are "disgruntled."

I submit to the judgment of an impartial public and independent profession, if the above does not warrant the conclusion that the organized medical profession, national and State, does not only regard it as a heresy that any member of the profession should not regard as holy and perfect the present organization, but that it is treason, punishable by the frown of their disapproval, that they should dare to criticise in print the plans and the method used to carry out their purposes; and even threaten with legal restraint the privilege possessed by every free American citizen to form with their fellows any society or organization that is not in conflict with the well-being of the commonwealth. But, fortunately, these individuals, being safe from any punishment, except their displeasure and exclusion from the charmed and sacred circle of the elect (which, happily for the individual, is of no material harm) can still continue to live and prosper. But the independent journal whose columns are open to all legitimate criticisms may not be able to survive the concerted onslaught of chartered monopolies, and may have their life crushed out by the chariot wheels of the journalistic Juggernaut of the A. M. A.

As Drs. Lyle and Olmsted chose, or their friends for them, to express themselves in the daily paper on this subject, in order that the public might know there was another viewpoint in the matter I sent the following, which was published:

EDITORS MORNING NEWS:

You had some days ago commented on a proposed new Medical Society in Georgia, and criticisms of that movement by Dr. Olmsted, of Atlanta, and there was some also in another daily, by Dr. Lyle, of Augusta. They call those who are interested in the formation of the new society "disgruntled," and they deny that there is any connection between the State Society and the National Society. I can show from the proceedings of both societies that the whole scheme of amalgamation began with the National body, which changed its constitution, not in accord with the provisions of the then existing constitution; and that the State society did the same thing, in the same way, not constitutionally, but revolutionary. I can show, also, that the only way to stay in the State Association now, no matter how long the membership may have existed, is to join, first, a county society; and to be a member of the National Association can only be through the State Association.

I can show by verbiage of the new constitutions of both associations by inference, and I believe also, from their proceedings, that one of the main objects of this "organization" and amalgamation is to create a strong political and financial body, whose powers will be used to further such end, and not chiefly for truly scientific medical research and advancement.

If either of the above gentlemen, or any one else, wishes to try conclusions with me on these points, I toss them my glove.

Respectfully,

T. M. MCINTOSH.

Thomasville, Ga., May 26, 1906.

During my entire professional life I have been a member of the Medical Association of Georgia, and would have been to-day had not its constitution been changed unconstitutionally by its "Politico-Medical" leaders.

Respectfully yours,

T. M. McINTOSH.

*Thomasville, Ga., July 16, 1906.*

---

UNSALTED DIET IN SCARLET FEVER.

Dufour, Pater and a number of other physicians have been studying the effect of restriction of salt in the diet of scarlet-fever patients. They find that it is well tolerated and protects against kidney trouble as well as a strict milk diet. If there is a tendency to albuminuria at first, it soon subsides. The children all increased in weight on the mixed unsalted diet as soon as the fever declined. The general impression was that the course of the disease was shortened and the patients rendered more resistant to incurrent infections and complications. Guinon, in a discussion of the subject at the Paris Société des Hop., February 8, stated that he made a practice of putting scarlet-fever children on raw meat, as well as those with slow-healing ulcerations, suppurations and necrosis. The raw meat seemed to exert a remarkably favorable action from the first, especially in the ulcerations that follow the phlegmons of scarlet fever or the necrosis of the palate. The so-called "unsalted diet" is not altogether without salt. A small, carefully measured amount is allowed and its elimination supervised.—*Journal A. M. A., March 10.*

---

IT is remarkable how frequently a purulent pericarditis may exist without causing many or severe symptoms. Never neglect an examination of the cardiac area, therefore, in cases of suspected sepsis.—*American Journal of Surgery.*

---

THE best site for an urgent tracheotomy is through the crico-thyroid membrane. To hold the opening apart a couple of hair-pins bent at the end may be used as retractors.—*American Journal of Surgery.*

